# Patterns of exposure to SARS-CoV-2 carriers manifest multiscale association between urban landscape morphology and human activity

**DOI:** 10.1038/s41598-021-01257-8

**Published:** 2021-11-11

**Authors:** Gabriel I. Cotlier, Yoav Lehahn, Doron Chelouche

**Affiliations:** 1 Haifa Center for Theoretical Physics and Astrophysics (HCTPA), The Data Science Research Center (DSRC), University of Haifa, Haifa, 3498838 Israel; 2 Department of Marine Geosciences, Charney School of Marine Sciences, University of Haifa, Haifa, 3498838 Israel

**Keywords:** Ecological epidemiology, Urban ecology

## Abstract

The outbreak of the Coronavirus disease 2019 (COVID-19), and the drastic measures taken to mitigate its spread through imposed social distancing, have brought forward the need to better understand the underlying factors controlling spatial distribution of human activities promoting disease transmission. Focusing on results from 17,250 epidemiological investigations performed during early stages of the pandemic outbreak in Israel, we show that the distribution of carriers of the severe acute respiratory syndrome coronavirus-2 (SARS-CoV-2), which causes COVID-19, is spatially correlated with two satellite-derived surface metrics: night light intensity and landscape patchiness, the latter being a measure to the urban landscape’s scale-dependent spatial heterogeneity. We find that exposure to SARS-CoV-2 carriers was significantly more likely to occur in “patchy” parts of the city, where the urban landscape is characterized by high levels of spatial heterogeneity at relatively small, tens of meters scales. We suggest that this spatial association reflects a scale-dependent constraint imposed by the city’s morphology on the cumulative behavior of the people inhabiting it. The presented results shed light on the complex interrelationships between humans and the urban landscape in which they live and interact, and open new avenues for implementation of multi-satellite data in large scale modeling of phenomena centered in urban environments.

## Introduction

More than 50% of the world’s population presently lives in cities, with an estimate forecast of the urban population in 2050 being around 10 billion^1^, making human health and well-being increasingly dependent on the dynamics of the urban environment. Current demographic trends, driven by rapid urbanization, migration flows, deforestation, climate change, inequality, and political instability have affected the dynamics of infectious diseases, and catalyzed the transmission of epidemic outcracks at global scale^2^. The increase in urbanization can function as an incubator for the spread of emerging zoonotic disease and epidemics^3^, and intra-urban crowding tends to promote the spread of infectious diseases^4^. The increase of urban agglomeration^5^, leading to a globally connected high population density, reinforces the need to better understand the possible impact of spatial changes in urban landscape on public health and in particular on the spread of diseases^6^.

Epidemiology and its association with spatial patterns of landscape and human activity have received much attention with the recent outbreak of the severe acute respiratory syndrome coronavirus 2 (SARS-CoV-2) pandemic, which causes the corona virus disease (COVID-19). Spatial data of COVID-19 incidence rates at county-level across the US, reveal a strong positive relationship with income inequality^7^. Network connectivity approach was applied to VIIRS and census data to quantify spatiotemporal evolution of SARS-CoV-2 epidemic propagation in the US; the findings indicate that spatiotemporal evolution of the epidemic may be quantified by analyzing transportation networks of different connectivity levels^8^. Satellite nighttime light (NTL) radiance and Air Quality Index data, although not without their associated challenges^9^, have been used to characterize spatiotemporal changes of human activity before and during the pandemic in China, showing that NTL radiance increased in residential areas and decreased in commercial centers after government lockdown was imposed, while transportation and public facilities seem to remain unaltered^10,11^. Employing mobile data tracking, a large increase in the daily usage of urban green areas by pedestrians and cyclists, who aim to preserve social distancing, was found in Oslo, Norway^12^. A body of research employing satellite observations has focused on analyzing the impact of SARS-CoV-2 epidemic restrictions on mobility and industry often showing reduction in anthropogenic emissions and improvement in air quality^13,14^.

Following the large body of work on the spatial aspects of disease spread in general, and of COVID-19 in particular, here we address the important, yet poorly investigated question of whether predictable relationships can be found between urban landscape, human activity, and patterns of exposure to SARS-CoV-2. Specifically, we hypothesize that patterns of heterogeneity in the urban landscape impose spatially varying constraints on interpersonal proximity and crowding conditions, thus affecting the potential for the spread of COVID-19. To test this hypothesis, we investigate the spatial association between patterns of exposure to SARS-CoV-2 carriers, with satellite-based landscape-heterogeneity measure, and nightlight intensity data. As a case study we focus on Israel, highlighting a selected sample of 30 cities, during a one-month period of intense COVID-19 outbreak between March and April 2020. We further explore in some detail the largest conurbation in Israel, named Gush Dan, which includes the metropolitan area of Tel-Aviv and is home to about 50% of Israel’s population.

## Results

Characterizing the spatial association between patterns of exposure to SARS-CoV-2 and urban landscape heterogeneity, requires an effective representation of the two components. According to the “patch-mosaic model”, landscape is composed of a structure of discrete units (or patches) assumed to have a relative inner uniformity and sharply defined boundaries which conform mosaics (or aggregation patches)^15,16^. Patchiness measures have long been used to characterize natural landscapes^17–19^, as well as for studying urban environments^20–22^. In order to accurately capture landscape heterogeneity across multiple scales, the definition of measures based on a continuous signal enables to capture the potential loss of information resulting from dividing the landscape into separate categorical units^23–25^. Several continuous spatial indices^26^ were developed to analyze urban landscape heterogeneity, including approaches such as spatial autocorrelation^27^, Fourier analysis^28^, and wavelet transform^29^ among others. Here, for grasping landscape heterogeneity across scales while reducing potential loss of information^30^ we apply a surface-based metric—the landscape patchiness index (LPI)—based upon scale variance model (see details in the Methods section). In general, high LPI values represent more "patchy" landscapes, with substantial variance at small spatial scales. In the data shown here, 95% of the LPI values range between -1.6 to -0.4.

**Figure 1:**
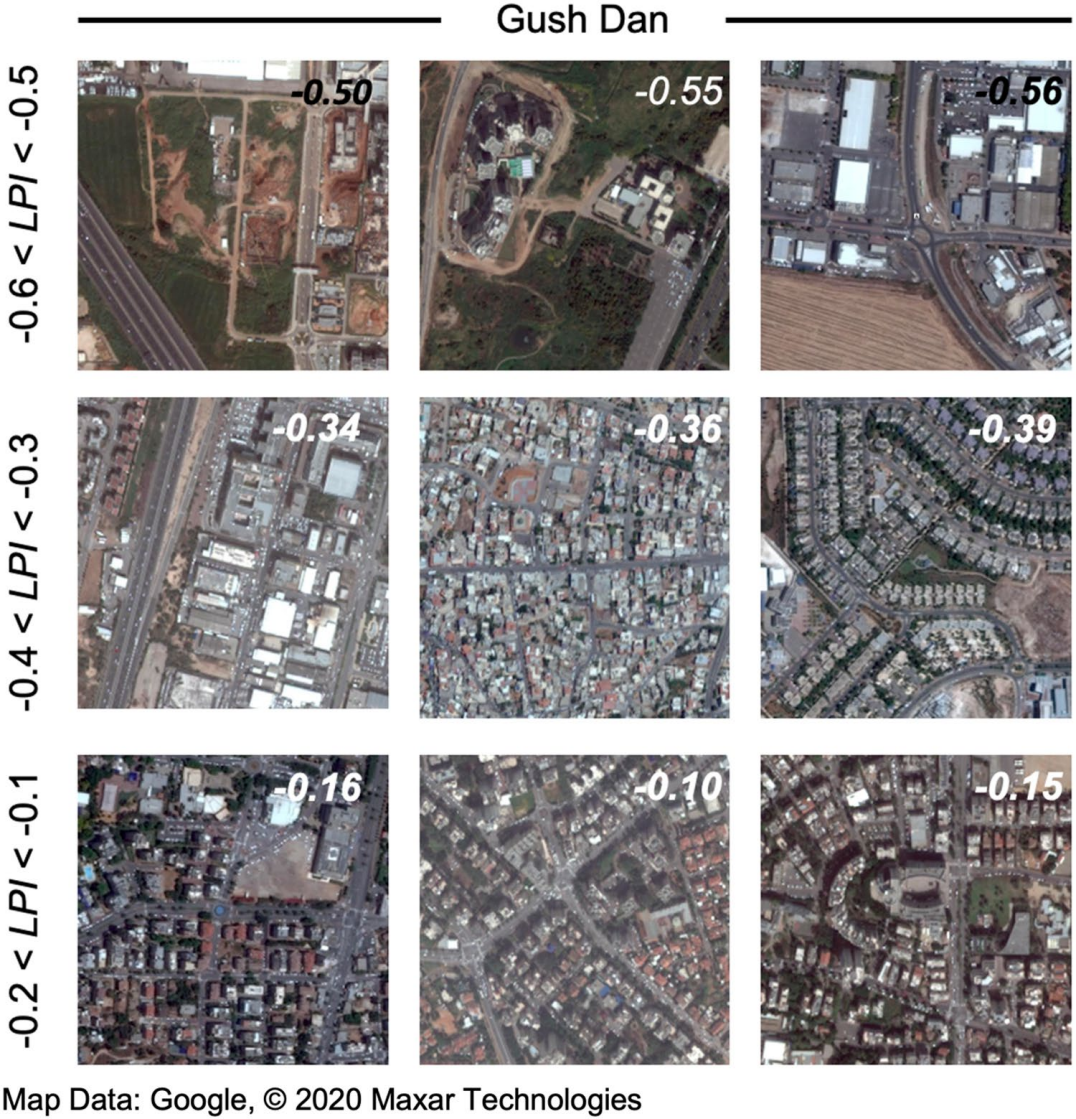
Examples for variations in urban landscape, as observed in Google map satellite images of cities in the Gush Dan District, across different ranges of LPI. Figure created using QGIS ^31^. For data sources of this figure see “Methods” section, subsection “Satellite data”.

The ability of the LPI to characterize multi-scale spatial patterns in the urban landscape, is exemplified by comparing randomly selected 480 m by 480 m Landsat images of urban areas characterized by different LPI levels (Fig. 1). Higher values of LPI, associated with high variance on small scales are often characteristic of highly developed urban regions, with relatively narrow streets in between the buildings, whereas lower values of LPI imply smoother landscapes, with less variance on small scales (compare lower and upper panels in Fig. 1, respectively). Note that the observed patchiness may also depend on seasonal effects (e.g., the presence of vegetation or lack thereof), as well as on the illumination angle of a non-smooth terrain (e.g., the casting of shadows by buildings, hills, and creeks). We find that although patchiness values are wavelength-dependent, the trends obtained here are robust.

Complementary information on the spatial characteristic of the urban landscape and its expression in patterns of human activity is obtained by the NTL. The NTL is commonly used in urban studies, and has been associated with socioeconomic dynamics^32^, population density^33^, urbanization^34^, crime analysis^35^, migrations^36^, military conflicts^37^, spread of epidemics^8^ identification of commercial areas^38^, and tourism^39^, constituting a good predictor of the magnitude of human activity.

**Figure 2:**
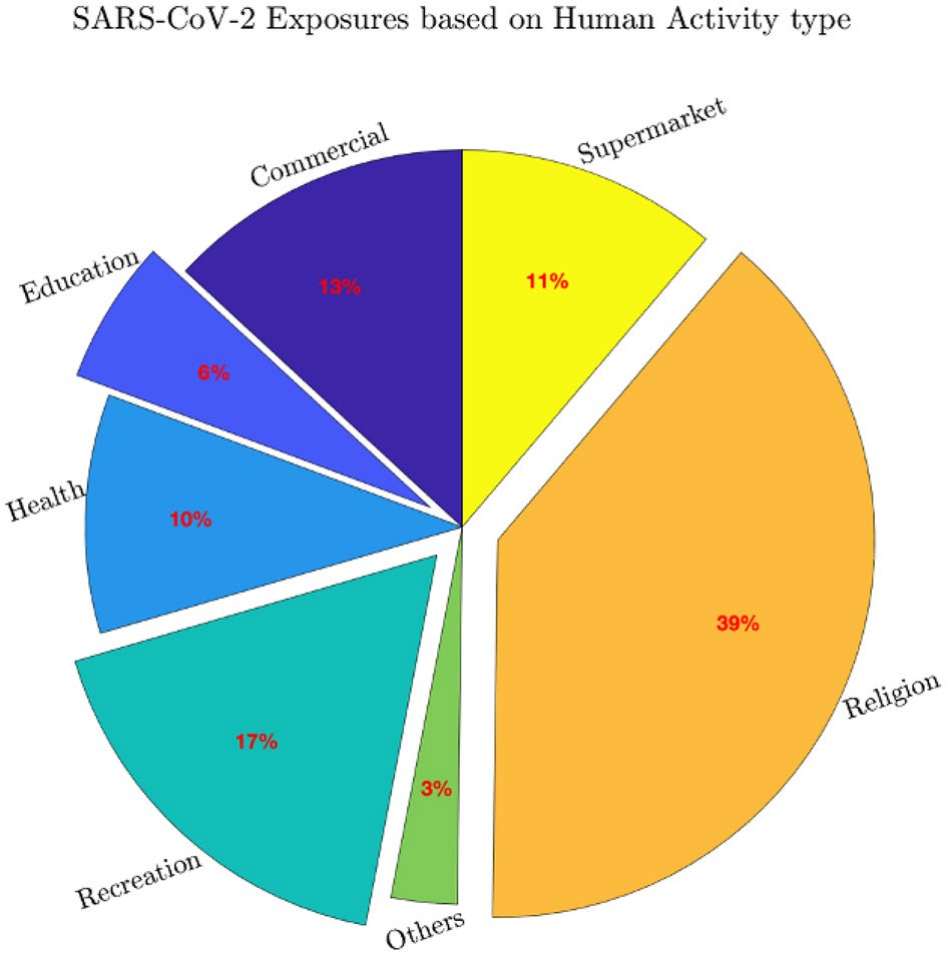
Pie chart describing the classification of RLSCs into human activity types.

Spatial characteristics of reported locations of SARS-CoV-2 carriers (RLSC) between 10/03/2020 and 14/04/2020 corresponding to the initial wave of COVID-19 were analyzed from the public spatiotemporal database compiled by the Israeli Ministry of Health^40^. The database consists of the results of epidemiological investigations, showing known locations of SARS-CoV-2 carriers up to two weeks prior to their diagnosis (after being diagnosed SARS-CoV-2 carriers have been immediately isolated). For the period covered by this study, the database consists of 17,250 reported RLSCs. To characterize the RLSC locations, we classify them into 7 categories of human activity according to the information provided in the epidemiological investigation, based on a randomly selected sub-sample of 10% of the total RLSC data set (Fig. 2). The largest amount (39%) of RLSCs were categorized as religion-related (including synagogues, Jewish religious studding centers, traditional baths, etc.); followed by recreational venues (17%, including culture establishments, restaurants, bars, coffee shops, sports centers, youth clubs, playgrounds, etc.); commercial grounds (13%, including different kinds of shops such as cloth, food, computers, cellphones, etc., as well as commercial services); supermarkets and grocery stores (11%, including small local to large chains); health facilities, (10% including clinics, hospitals, pharmacies, etc.); and education establishments (6%, including from kindergartens to universities, language school, research centers, etc.).

**Figure 3:**
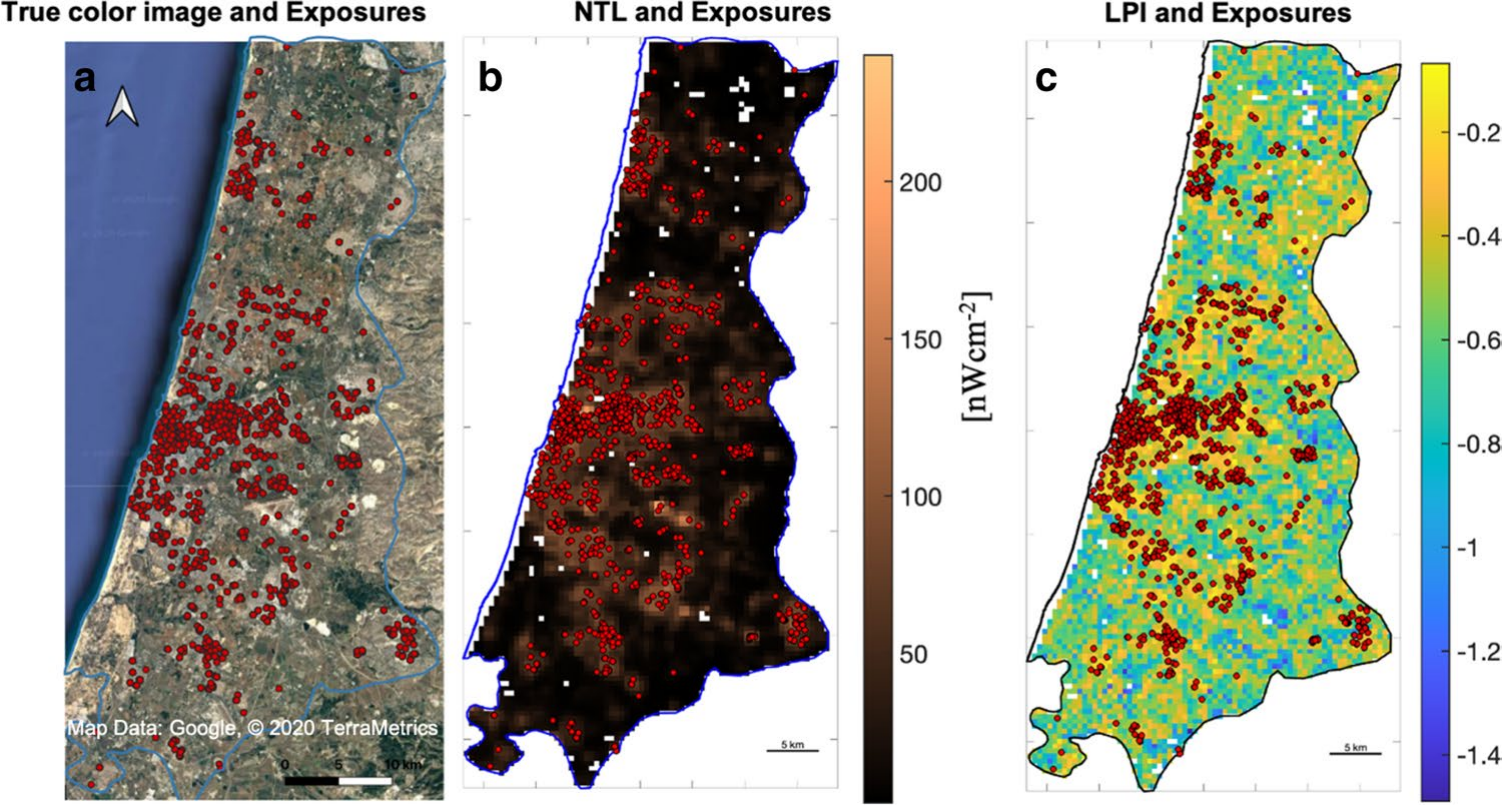
Spatial distribution of RLSC in Gush Dan District. RLSC in Gush Dan (red points), overlaid on **(a)** a Google maps satellite image. Blue line delineates the borders of Gush Dan district; **(b)** NTL map; **(c)** LPI map. Note that RLSCs preferentially occur in regions with high LPI values, which correspond to regions with high NTL values. Panel 3a created using QGIS^31^, panels **(b,c)** created using MATLAB^43^ (for more information see “Method” section, subsection “Satellite data”).

To further investigate the factors underlying spatial distribution of RLSC we compare it with the spatial characteristics of surface measures extracted from satellite data. We first focus our analysis on the Gush Dan District, which is a densely populated area including Tel Aviv city and its metropolitan area. It has total population of approximately about 4 million residents, comprising about 44% of the population of Israel^41^ (Fig. 3). The dependence of RLSC distribution on landscape morphology can be qualitatively observed in a true colors satellite image, which emphasizes the fact that RLSC data points are mostly found in highly developed urban areas (Fig. 3a). In agreement with that, RLSCs are mostly found in areas of high NTL levels, with mean values of 72.166 $\pm 0.574$ (nWcm^-2^). For comparison, the average NTL level in regions with no reported RLSCs is 19.78 $\pm 0.273$ (nWcm^-2^) (Fig. 3b). Interestingly, a tracer to RLSC distribution in space is also provided by the LPI, which is characterized by average values of -0.343 $\pm 0.007$ and -0.522 $\pm 0.002$, for grid cells containing and not containing RLSC data points, respectively (Fig. 3c).

**Figure 4:**
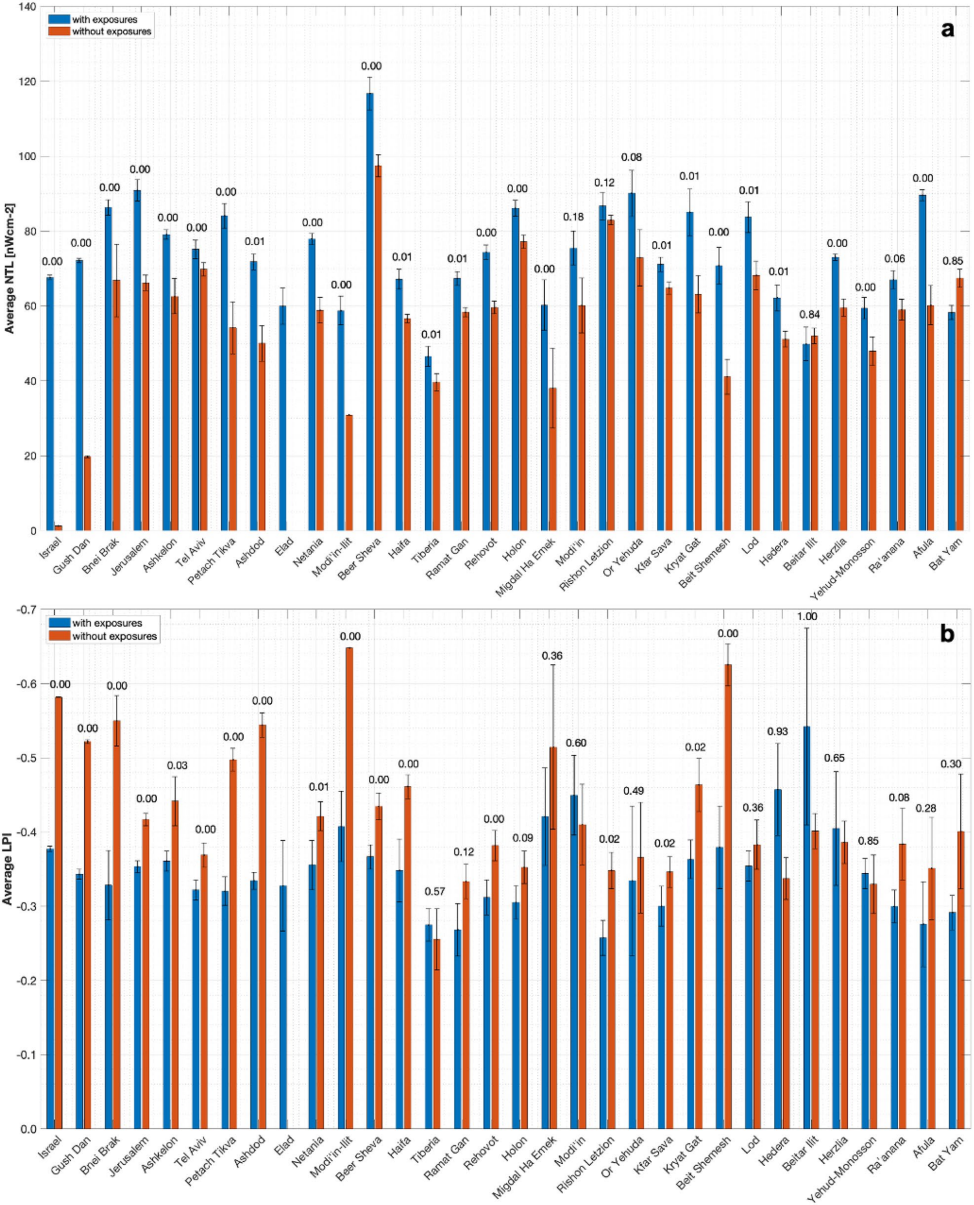
NTL and LPI statistics for our sample. Bar plot showing the mean of (a) NTL and (b) LPI in grid cells with exposures (blue bars) and without exposures (red bars) for the 30 cities with more than 100 reported RLSC cases. For comparison we also present the statistics for the full sample of cities, as well as that for the Gush Dan conurbation. Also denoted above the bars for each city/region are the p-values ($0\le \mathrm{p}\le 1$) with which our null hypothesis—that the average NTL/LPI values for RLSC and for regions with no reported exposures are drawn from the same distribution—holds. Note that in almost all of the cities both NTL and LPI values are significantly higher at locations with finite RLSC values.

Following this relatively coarse analysis, which largely reflects distinction between urban and non-urban landscapes, we next probe the landscape patterns underlying RLSC spatial distribution at the city scale. To this end, we first compare average NTL and LPI values at locations with RLSCs to those values at locations without RLSC. We do so for the 30 cities reported by the Israeli Ministry of Health to have more than 100 RLSCs (Fig. 4). We find that average LPI and NTL values are significantly higher at locations with reported RLSCs in 77% and 90% of the cities, respectively. This demonstrates that LPI, like NTL, can be used as a tracer of intense human activity within the urban landscape.

**Figure 5:**
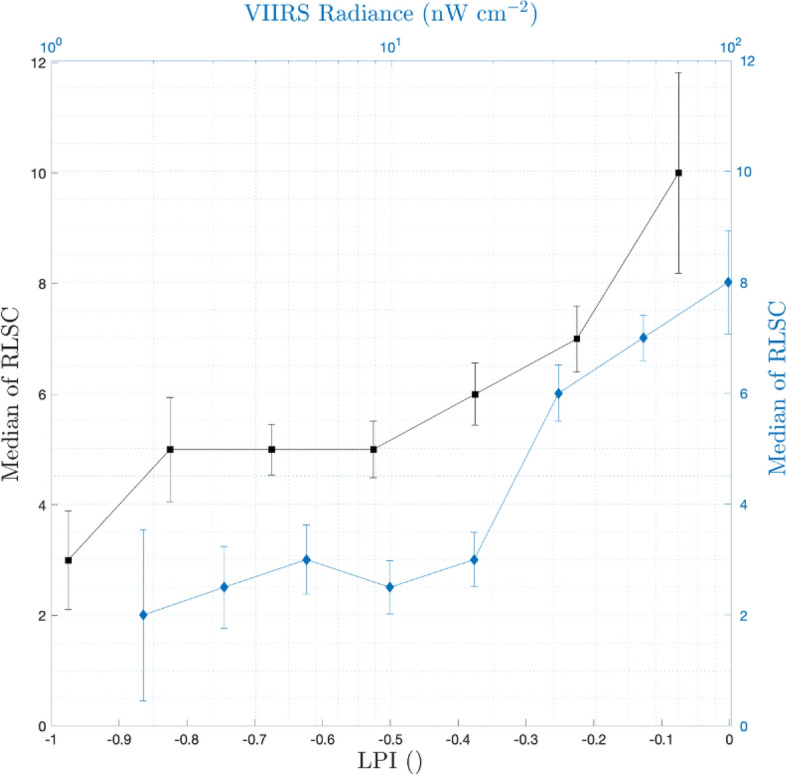
Correlation between RLSC and NTL (blue curve) and LPI (black curve). Dots and error-bars denote the median and standard deviation in each bin, respectively. Note the qualitatively similar behavior for the NTL and LPI statistics demonstrating the commonalities between LPI and NTL as tracers of human activity and landscape development.

The association between the RLSC and the spatial characteristic of the urban landscape is emphasized when considering the median RLSC values for locations as a function of LPI/NTL (the LPI/NTL range is divided into bins, and the median RLSC is calculated for all locations corresponding to those bins). Results for the entire area covered by the Ministry of Health reports are shown in Fig. 5. Evidently, similar behavior is found for the NTL and for the LPI, whereby higher values, which correspond to more developed areas, are associated with a higher (median) RLSC values. Interestingly, there is little dependence of the median RLSC on the LPI or the NTL for low values in those properties. In contrast, a much steeper relation is observed for higher values of NTL, LPI, and RLSCs. The similar behavior of NTL and LPI statistics further demonstrates that LPI, like NTL, is a tracer of human activity and development.

## Conclusions

Using an integrated dataset comprised of satellite observations and results from epidemiological investigations, we explore the interrelationships between spatial characteristics of urban landscape, human activity, and distribution patterns of reported locations of SARS-CoV-2 carriers, or RLSC, in a highly populated region in the state of Israel. Our results show that at least for the time and location covered in this study, satellite-based levels of NTL and LPI are statistically significant measures of the likelihood of a given region within the urban area to host a RLSC. While linkages between NTL and different aspects of human activity were previously investigated in numerous studies ^8,32––39^, the association between spatial patterns of human activity, and satellite-derived urban landscape heterogeneity, as expressed here by the distribution of RLSC and levels of LPI, respectively, to the best of our knowledge is shown here for the first time.

This research strongly relies on epidemiological investigations that are meant to track trajectories SARS-CoV-2 carriers prior to being diagnosed, in order to identify potential cases of exposure and, ultimately, stop the chain of infection. Spatial distribution patterns of RLSC emerging from the investigation are therefor most likely linked to patterns of human agglomeration and reflect pathways of disease spread. Accordingly, the spatial association between RLSC and NTL can be explained by the fact that higher values of the NTL simply represent areas of intense human activity, where encounters between people are more frequent.

While NTL values are directly linked to the level of human activity, LPI captures the spatial characteristics of the urban landscape in which the human activity takes place. We suggest that the spatial association between LPI and RLSC reflects a fundamental scale-dependent constraint imposed by the morphology of the city’s landscape on the cumulative behavior of the people inhabiting it. Accordingly, patchy (i.e. high LPI) sections of the city, in which landscape variance becomes important at small spatial scales (a few to tens of meters) that resonate with typical scales of human interactions, are more prone to human agglomeration and crowding, and thus to disease spread. In contrast, in areas of homogeneous landscape, with relatively small contribution to the overall landscape variance from small scale patterns, encounters between people are, in general, less frequent. Consequently, as observed here, events of exposure to SARS-CoV-2 carriers are significantly more likely to occur in patchy parts of the city.

In addition to improving our understanding on the interrelationship between humans and the urban landscape in which they live and interact, the unique observations presented in this work open new avenues for implementing multi-satellite data, as the ones used here, in large scale modeling of phenomena centered in urban environments. Specifically, the significant relationship between LPI and RLSC allows incorporating the effect of urban landscape morphology, as traced by satellite data, in epidemiological models. Such implementation is likely to improve human society’s ability to predict, and potentially mitigate, the spread of diseases as the COVID-19.

## Methods

### Epidemiological data

Spatial characteristics of reported locations of SARS-CoV-2 carriers (RLSC) between 10/03/2020 and 14/04/2020 were analyzed from the public spatiotemporal database compiled by the Israeli Ministry of Health^40^. The database consists of the results of epidemiological investigations, conducted on positively diagnosed SARS-CoV-2 patients. The database was updated on a daily basis, with results available online for a 2-week period. From this database we used georeferenced information on locations of SARS-CoV-2 carriers to two weeks prior to their diagnosis.

### Extraction of the landscape patchiness index (LPI)

We introduce a modified version of an analytical method based on the so-called “geographic variance” or “scale variance” approach^42,44,45^ for describing the formation and spatial dynamics of landscape heterogeneity across scales^46^. Employing a nested hierarchy of levels across multiple scale enables to capture each level’s independent contribution to the total system’s variance. Different versions of this method haven been applied in different disciplines such as oceanography^47^, and economic geography^48^. Here we employed a mosaic composed of three Landsat 8 Level-2 surface reflectance images downloaded from USGS Earth Explorer^49^, corresponding to the green visible range (0.525–0.600 µm) and having a native spatial resolution scale, ${\mathrm{l}}_{0}=30$ m. The scale-dependent variance, ${\upsigma }_{\mathrm{n}}^{2}$ is calculated within sub-image tiles of size ${\mathrm{l}}_{\mathrm{n}}={2}^{\mathrm{n}}{\mathrm{l}}_{0}$, with n = 1,2,3,4 so that the maximum tile-size considered is ${\mathrm{l}}_{\mathrm{max}}=480$ m, which is comparable to the VIIRS night-light image resolution^50^. The calculation of the LPI proceeds as follows: the scale-dependent mean variance, $\langle {\upsigma }_{\mathrm{n}}^{2}\rangle$ is obtained by averaging over ${\upsigma }_{\mathrm{n}}^{2}$-values for all the sub-image tiles of size ${\mathrm{l}}_{\mathrm{n}}$ that are within in a given tile of size ${\mathrm{l}}_{\mathrm{max}}$. A powerlaw fit to the scale-dependent variance, $\langle {\upsigma }_{\mathrm{n}}^{2}\rangle \propto {\mathrm{n}}^{-\mathrm{p}}$ is then obtained, where $\mathrm{p}$ corresponds to the LPI at that location. Higher values of $\mathrm{p}$ imply substantial variance on small scales, which often characterizes developed regions, where structures are of order human scales, which are ${\ll \mathrm{l}}_{\mathrm{max}}$ (see examples in Fig. 1). Conversely, lower values of $\mathrm{p}$ imply smoother landscapes.

**Figure 6:**
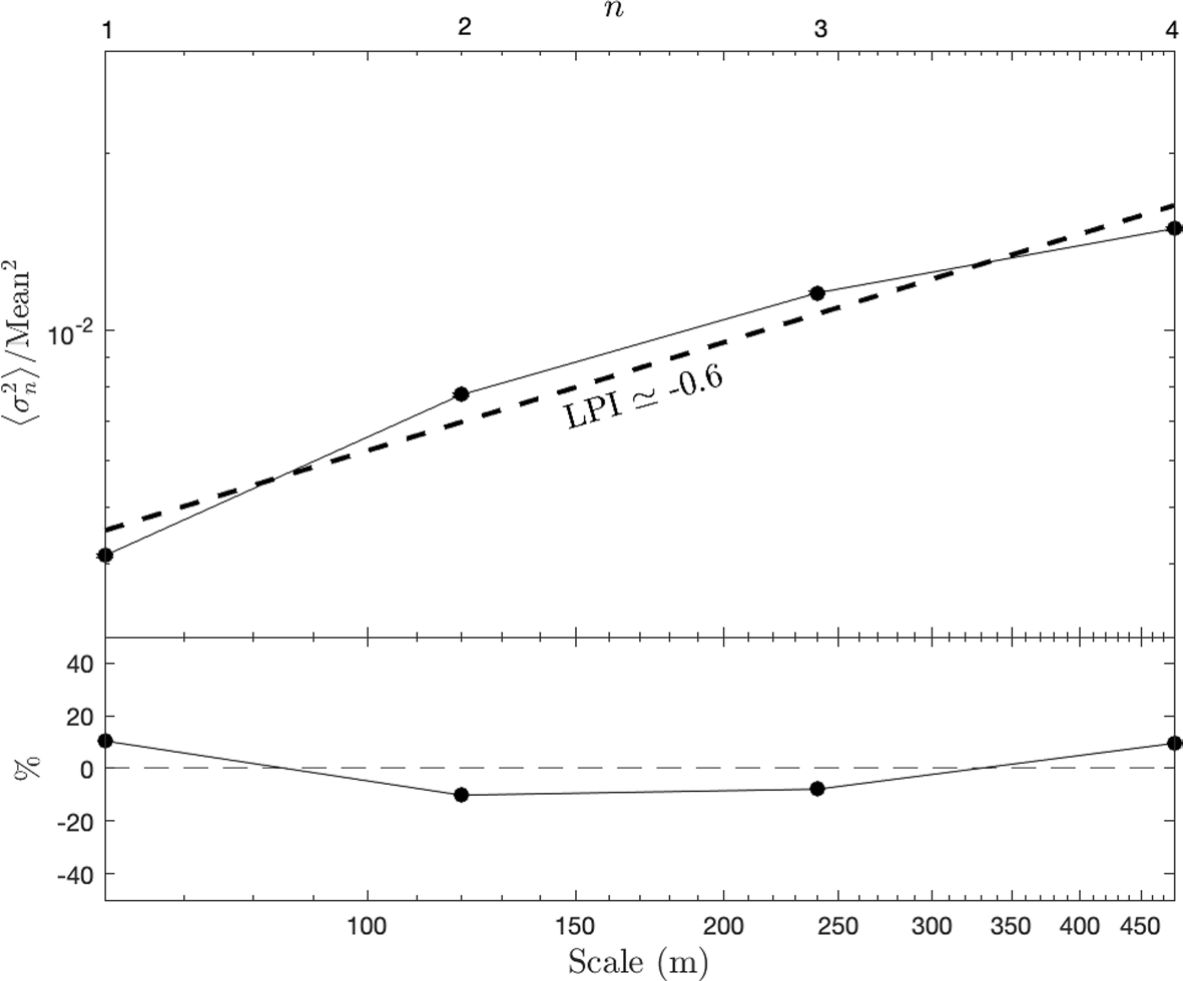
The average scale-dependent variance and the definition of the LPI. Upper panel shows the scale-dependent variance (normalized by the square mean of the map) when averaged over all tiles comprising our region of study. A monotonically increasing variance with scale is clearly seen, which is well traced by a powerlaw behavior (dashed line) with an average LPI of -0.6. Lower panel shows deviations between the data and the best-fit powerlaw model, which are at the ≤ 10%-level.

The above approach, whereby a single powerlaw model is fit to the scale-dependent variance, assumes no preferred length-scale over the scale-range probed. This assumption cannot be verified on a tile-by-tile basis but can be justified by calculating the average scale-dependent variance for the entire region and fitting a powerlaw model to the ensemble average. Specifically, deviations from a powerlaw fit over the scale-range probed are typically less than 10% (Fig. 6). This combined with the fact that spatial patterns are clearly discernible in the LPI map, and that good correlations are observed between it and independent datasets, support the powerlaw model employed here. The study of the small deviations from a powerlaw behavior is beyond the scope of the present work.

### Satellite data

The LPI is derived from Landsat 8 Level-2 surface reflectance images of the 31 of July 2017 downloaded from USGS Earth Explorer^49^, corresponding to the green visible range (0.525–0.600 µm) with 30 m of spatial resolution. Nighttime light intensity is derived from monthly average radiance composite data of July 2017 from Suomi National Polar-orbiting Partnership (SNPP) VIIRS Day-Night Band (DBN) downloaded from Google Earth Engine^51^ with an estimated spatial resolution of ${15\times 15\mathrm{ asec}}^{2}$, which roughly corresponds to $460 \times 460 {\mathrm{m}}^{2}$ for the latitude of study (Fig. 3b). The true colors satellite images shown in Fig. 1 and 3a were downloaded from Google maps (https://www.google.com/maps/).

## Data Availability

Israeli Ministry of Health geodatabase is freely available at https://imoh.maps.arcgis.com/apps/webappviewer/index.html?id=20ded58639ff4d47a2e2e36af464c36e&locale=he&/ Landsat 8 data is freely available from USGS Earth Explorer at https://earthexplorer.usgs.gov VIIRS data is freely available at https://developers.google.com/earth-engine/datasets/catalog/NOAA_VIIRS_DNB_MONTHLY_V1_VCMCFG.
